# New Approaches To Identify Urine and Hair Adulteration Attempts in Forensic Toxicology: A Proof-of-Concept Study Using a Proteomics Approach Based on Liquid Chromatography–Mass Spectrometry (LC-MS)

**DOI:** 10.1021/acs.analchem.5c03096

**Published:** 2025-08-05

**Authors:** Tom D. Schneider, Tina M. Binz, Thomas Kraemer, Andrea E. Steuer

**Affiliations:** Department of Forensic Pharmacology & Toxicology, Zurich Institute of Forensic Medicine, 87757 University of Zurich, 8057 Zurich, Switzerland

## Abstract

Urine and hair are among the primary biological matrices used for drug and abstinence testing in clinical, forensic, and antidoping settings. A persistent challenge in such analyses is the adulteration of samples: through dilution, substitution, or chemical modification, aimed at concealing the presence of xenobiotics such as drugs of abuse, ethanol, or doping agents. In this study, we investigated whether chemical adulteration, specifically oxidative treatment with hydrogen peroxide (H_2_O_2_), induces detectable and characteristic changes in the proteomes of urine and hair samples. Using a bottom-up proteomics approach involving LC-HR-MS/MS with data-dependent acquisition, we compared urine samples before and after treatment (10% H_2_O_2_) and untreated hair samples with those exposed to increasing concentrations of hydrogen peroxide (3%, 6%, 9%, and 12%). Data are available via ProteomeXchange with the identifier PXD064208. We identified distinct peptides, including oxidatively modified forms, that were exclusively present in either untreated or chemically treated groups. In hair samples, the appearance of some of those peptides was dependent on the peroxide concentration. Peptides detectable only after oxidative exposure were of particular interest, as they appeared to be nonphysiological and specific to the adulteration process. These species serve as candidate biomarkers for indirect detection of sample manipulation or for assessing the integrity of compromised samples. The extraction and characterization of these potential marker peptides constitute the primary outcomes of this study. These findings should lay the groundwork for further validation and the development of proteomic methods aimed at enhancing the reliability of drug testing and sample authenticity assessment in forensic and antidoping contexts.

## Graphical abstract

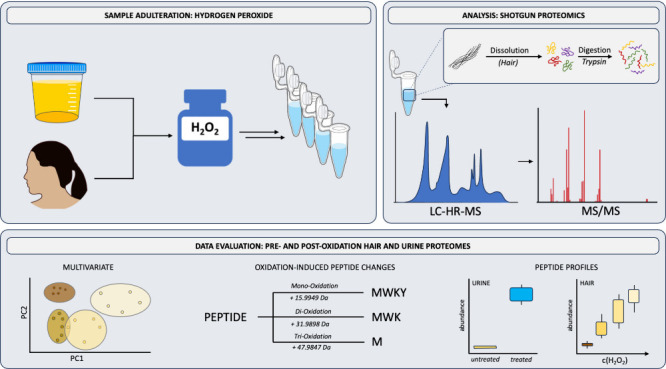

## Introduction

Substance abuse still represents a significant public health issue worldwide. In the context of activities that require a high level of attention and concentration, such as driving, certain workplaces, or the military, monitoring of substance use/misuse plays a critical role.^1–6^ Various matrices, nowadays mainly urine and hair followed by blood or saliva, can be used to exclude intake of ethanol, drugs of abuse (DOA), or prescription drugs, typically within country-specific abstinence control programs. To avoid severe legal or economic consequences potentially associated with a positive drug testing result, drug users may be tempted to manipulate their samples to deliberately “remove” drugs so that they cannot be detected. Samples are commonly defined as negative when drugs cannot be detected or, more often, if drug concentrations fall below a certain specified cutoff value. As such, deliberately decreasing the drug concentration below the cutoff may already be sufficient to achieve a negative test result. There are numerous strategies to manipulate a sample in order to present it as drug-free or with too low a concentration of substances. Even commercially available products exist. For urine samples, dilution, substitution, and chemical adulteration represent the most common strategies.^5,7,8^ Hair manipulation includes forced washing-out attempts or chemical treatment, respectively. Regarding hair, it remains impossible to analytically differentiate between usual cosmetic hair treatment, e.g., bleaching, and deliberate manipulation through oxidation by hydrogen peroxide (H_2_O_2_).

Next to the constant challenges for adequate drug testing arising from the highly dynamic drug market, routine screening for adulteration attempts would be necessary. Drug and adulteration testing methods not only require high sensitivity and specificity but also should be economical and fast enough to allow high-throughput workflows if necessary. Therefore, dipstick tests or automatic testing by autoanalyzers still represents the standard procedures but can be associated with high numbers of false positives and negatives.^5,9^ Progress has been made in recent years to evaluate alternative approaches to screen for sample manipulation attempts, as discussed recently by Wissenbach and Steuer.^10,11^ The focus was on small, endogenous compounds, evaluated as new validity or invalidity parameters^12–14^ or particular biomarkers of adulteration,^15–20^ mainly by liquid chromatography–mass spectrometry (LC-MS) approaches. Advances in bioassays were also evaluated, again with the aim of detecting endogenous analytes such as uric acid,^9,21,22^ but these were found to be comparatively inferior to LC-MS.^22^ Analysis of small endogenous molecules seems to represent the most straightforward approach in clinical and forensic toxicology given their similarity to DOAs in terms of molecular weights and analytical methodology. However, when it comes to untargeted approaches aiming at identifying new biomarkers, such metabolome-like approaches still suffer from insufficient identification power of the most promising analytical features.^17^ As such, the presumably most promising biomarkers to detect adulteration attempts remain unidentified and thus are unusable for routine analysis.

With proteomics, a large proportion of a specimen’s proteome can be analyzed quickly, and many potential biomarkers can be investigated simultaneously. Like metabolomic-based approaches, untargeted proteomics is considered a holistic approach in biomarker research. However, proteomics can be considered superior in identifying potential biomarkers, as the transition of ambiguous *m*/*z*-ratios into an identified (bio-) molecule is much more straightforward due to the combinatorial nature of protein sequences and predictable peptide fragmentation patterns. The human hair proteome is predominantly composed of keratins and keratin-associated proteins, reflecting structural specialization, whereas the human urine proteome comprises a diverse array of extracellular and intracellular fragments derived from plasma, kidney and urogenital tissue.^23–25^

The present proof-of-concept study aimed to assess the feasibility of analyzing proteins and peptides of human urine and hair samples by LC-MS to detect oxidative adulteration with H_2_O_2_.

## Materials and Methods

### Chemicals and Reagents

Ammonium bicarbonate (ABC), urea, tris­(2-carboxyethyl)­phosphine (TCEP), and iodoacetamide (IAA) were purchased from Sigma-Aldrich (Steinheim, DE). Tris-HCl (ultrapure) and QuBit protein assay reagents were from Invitrogen (Carlsbad, CA, USA, and Eugene, OR, US, respectively). Trypsin and dithiothreitol (DTT) were purchased from Thermo Scientific (Rockford, IL, US, and Vilnius, LT, respectively). iRT peptides were obtained from Biognosys (Schlieren, CH). Methanol (Optima LC/MS grade) was sourced from Fisher Scientific (Loughborough, UK), chloroform (Emsure analysis grade) from Merck (Darmstadt, DE), dimethyl sulfoxide (DMSO) from Thermo Scientific (Rockford, IL, US), and hydrogen peroxide solution (50% pure) from AppliChem (Darmstadt, DE). STAGE-Tips were manually produced in-house using Empore C18 material supplied by Supelco (Bellefonte, PA, US) and 200 μL tips. Total recovery vials from Waters (Milford, MA, USA) were used for LC-MS/MS analysis. Water was purified with a Millipore filtration unit, and HPLC grade methanol was obtained from Fluka (Buchs, Switzerland). All other chemicals used were from Merck (Zug, Switzerland) and were of the highest grade available.

### Urine and Hair Samples

Authentic human urine was collected from four different healthy volunteers and stored in polypropylene tubes at −20 °C until analysis. Hair samples of brunette and black were collected from seven different healthy volunteers (not matched to urine specimens) and stored at room temperature until analysis.

All volunteers provided written informed consent. According to Swiss ethics (Humanforschungsgesetz), no further ethical approval from the cantonal ethics commission is necessary if the research is not aiming to investigate diseases or functions of the human body, as has been the case in the current study. No use of drugs, medications, or other relevant xenobiotics was reported from any volunteer of the donated samples.

### Adulteration Procedure and Sample Preparation for Urine Samples

Urine samples were taken from four donors, and each individual’s sample was separated into two halves. The first half was left as is (untreated), while the second half was treated with 10% H_2_O_2_ (v/v) for 30 min. The (treated) urinary proteins were subsequently isolated by protein precipitation using a mixture of ice-cold chloroform and methanol (3:1, v/v). The precipitate that formed was washed and reconstituted in an aqueous 8 M urea solution. In-solution digestion was performed using a standard overnight in-solution digest with trypsin.^26^ Digestion was stopped by acidification, and the resulting peptides were cleaned and desalted using a standard STAGE tip protocol (C18 solid-phase stamps; wetting with 100% ACN; equilibration with 60% ACN + 0.1% formic acid; condition/wash with 3% ACN + 0.1% formic acid; elution with 60% ACN + 0.1% formic acid).^27^ A total of 5 μg of purified isolated peptides was injected.

### Adulteration Procedure and Sample Preparation for Hair Samples

Hair samples from seven donors were collected and separated into four aliquots of hair strands, respectively. Three pools were created from these seven donors and used as native samples (*n* = 3). The remaining three were treated with increasing concentrations of H_2_O_2_ solution (3%, 6%, 9%, and 12%, v/v) for 30 min, respectively. For 6% and 9% H_2_O_2_ treated samples, one volunteer’s sample was insufficient for further sample preparation and analysis. Subsequently, the hair matrix was chemically and physically dispersed as follows: hair samples were suspended in a solution of 8 M urea, 50 mM ammonium bicarbonate, and 200 mM tris­(2-carboxyethyl)­phosphine (TCEP), and the resulting suspension was treated with ultrasound and kinetically disrupted using a magnetic stirring bar at 1300 rpm for 4 h. Cysteines were carbamylated with an excess of iodoacetamide (IAA, 400 mM), and an aliquot of solubilized hair protein was diluted down to <1 M urea. Subsequently, in-solution digestion was performed using an overnight digest with trypsin.^26^ Digestion was stopped by acidification, and the resulting peptides were cleaned and desalted using a STAGE tip protocol (see above). A total of 5 μg of purified peptides was used for injection.

### HPLC–HRMS Analysis

Analysis was performed in random order on a Thermo Fisher Ultimate 3000 UHPLC system (Thermo Fisher Scientific, San Jose, CA) coupled to a high-resolution time-of-flight (TOF) instrument (TripleTOF 6600, Sciex, Concord, Ontario, Canada) system. The Thermo Fisher Ultimate 3000 UHPLC system was adapted to suit lower flow rates by replacing the preinstalled mixing chamber and sample loop with corresponding lower dead-volume counterparts. A dedicated LC-MS method was adapted to suit the analytical and experimental prerequisites under the already pre-existing instrumental infrastructure based on the works from Bian et al.^28–30^ A mixture of H_2_O, 3% DMSO, and 0.1% formic acid (v/v/v) served as mobile phase A while ACN with 3% DMSO and 0.1% formic acid (v/v/v) was used as mobile phase B. An LC gradient elution was performed using an Acclaim PepMap column (1.0 mm × 150 mm, 2 μm particle diameter) at 55 °C at a 50 μL/min flow rate over a 30 min gradient from 3% to 35% mobile phase B for urine samples and a 60 min gradient for hair samples. An increase to 95% mobile phase B followed as a column wash-out phase (10 min) and re-equilibration to starting conditions (3% mobile phase B) was executed over 5 min.

HRMS analysis was performed in positive ionization mode with a DuoSpray ion source at a resolving power (full width at half-maximum, fwhm at *m*/*z* 400) of 30 000 in MS^1^ and 30 000 in MS^2^ (high-resolution mode). Automated MS calibration was performed by repeated injection after every 10th sample of Ultramark 1621 calibrant solution via the LC system. The source conditions were set as follows: MS^1^ acquisition was performed over a selected mass range from 315 to 1850 *m*/*z* using an accumulation time of 200 msecs. Further, MS^2^ experiments were performed using data-dependent acquisition (DDA) under the following MS^2^ settings: mass range from *m*/*z* 100 to *m*/*z* 1500, accumulation time for each DDA scan of 100 ms, with rolling collision energy enabled 0 eV in high sensitivity mode. DDA criteria were set as follows: charge-state filter: + 2 to +5, dynamic background subtraction, 12 most intense ions from precursor MS^1^ scan with an intensity threshold of 500 cps, and exclusion time window of 30 s after 1 occurrence. The mass spectrometry proteomics data have been deposited to the ProteomeXchange Consortium via the PRIDE partner repository with the data set identifier PXD064208.^31^

### Data Processing and Statistics

Prior to data analysis, the acquired mass-spectral raw data files (.wiff) were converted to generic mzML files using MSconvert^32^ (v 3.0) and entered into the FragPipe^33^ (v 22.0) proteomics data pipeline. The human protein database entry Uniprot 9606, reviewed, including decoys and common contaminants, was used as a FASTA file for spectral matching. An “open” search workflow was run first via FragPipe in order to explore the “landscape” of observable modifications on the protein and peptide levels. MSFragger settings were set as follows: precursor mass tolerance, 20 ppm; fragment mass tolerance, 20 ppm; protein digestion (cleavage), enzymatic; clip N-term M yes; enzyme, strict trypsin. Variable modifications were allowed for methionine oxidations (+15.9949 Da, +31.9898 Da, +47.9847 Da), and N-terminal acetylation (+42.0106 Da); as well as fixed modifications for cysteine iodoacetamidation (+57.02146 Da). FragPipe validation tools (running crystal-C, PSM validation, protein inference via ProteinProphet) and an FDR filter were enabled. MS^1^ quantification in FragPipe was conducted using IonQuant and label-free quantification (LFQ), with match between runs and normalization of intensity across runs enabled. Protein and peptide abundances were further evaluated and visualized using Prism v 9. Raw results from FragPipe were variance-filtered using interquantile range, filtered by low abundance (close to baseline, < 500 cps) and log_10_-transformed. Statistical and multivariate analysis (ANOVA, *t* test, principal component analysis, PCA, and partial least-squares discriminant analysis, PLS-DA and hierarchical clustering) was carried out using R (v 4.2) with these packages: ggplot2, limma, stats, ropls, and pheatmap.

Multivariate analysis results were screened for prospective peptide candidates by the following criteria: (a) undetectable in either treated or untreated condition, and only results where the lowest groupwise median abundance exceeded 10^5^ cps were retained; (b) by significance of differences (paired *t* test, *p* < 0.001 or ANOVA, respectively) between treatment conditions; (c) coefficient of variation (CV) of <150% (for urinary peptides) or <250% (for hair peptides); (d) (non-)­detectability in all samples from the same sample treatment cohort (complete analytical coverage across samples per condition).

## Results and Discussion

### Induced Changes to Peptides through Oxidative Treatment

Modifications on the peptide level, especially oxidation of specific sites/amino acids, are very well described in the literature and are linked to oxygen exposure and aging and/or ex vivo sample decaying processes.^34,35^ Methionine is commonly found to be prone to oxidation and is rarely found exclusively nonoxidized in bottom-up proteomics experiments. Yet, the extent, i.e., frequency and abundance of oxidized methionine and other amino acids, plays an essential role in assessing a sample. This could also be used for forensic questions regarding sample adulteration: The key aspect of this study lies in identifying (modified) peptides that, when detectable or absent, are indicative of sample adulteration (or indicative of no sample adulteration, respectively). The harsh oxidative treatment using H_2_O_2_ introduced many changes within both the proteomes of urine and hair samples. A general occurrence of higher oxidation states of included AA modification sites was observed and was dependent on the extent of H_2_O_2_-treatment in hair samples (linear relationship between these and the used H_2_O_2_ concentrations; data not shown). Data obtained from conducting an open search via ProteinProphet of both urine and hair proteomes resulted in certain peptide/amino acid modifications of interest, based on their overall no. of appearances: mono oxidation (+15.9949 Da) of methionine, tryptophan, lysine, and tyrosine, 2-fold oxidation (+31.9898 Da) of methionine, tryptophan, and lysine, and triple oxidation (+47.9847 Da) of methionine.

### Oxidative Changes on the Urine Proteome: Selection of Potential Adulteration Markers

A volcano plot analysis (Figure 1A) revealed a total number of 177 peptides that exhibited a significant increase after oxidation with H_2_O_2_. In comparison, 151 peptides showed a significant decrease instead, and a total of 415 did not show a significant change. Hierarchical clustering based on *t* test scores of observed changes (Figure 1B) highlights the characteristic differences between treated urine samples and allows for a preselection of prospective peptides.

**Figure 1:**
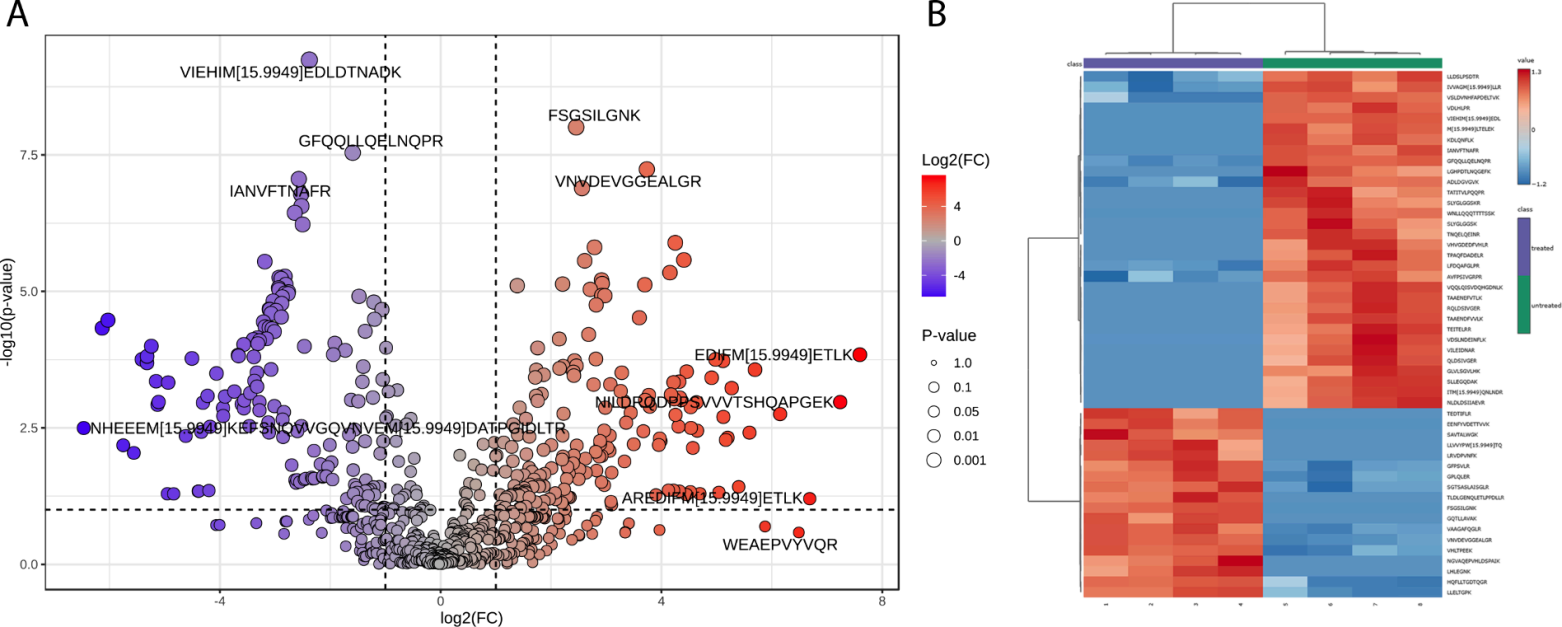
(A) Volcano plot of urine samples treated with hydrogen peroxide. The fold-change threshold is set to 2.0. *p*-value threshold of <0.01 (false discovery rate). Fold-change direction: treated over native. Number of sig. increased peptides 177, sig. decreased peptides 151, and nonsignificantly changed peptides 415. (B) Hierarchical clustering (“heat-map”) representing the top 50 peptides for classification according to *t* test scores with sample class clustering enabled. Color gradient (blue to red) represents log_10_-transformed peptide abundances (low to high).

By further dissecting these analytical findings, peptides carrying oxidized modifications were found to be of the most promising nature to be used as possible biomarkers for oxidative adulteration attempts: certain peptides could only be detected in samples that were treated with H_2_O_2_ and remained fully undetectable in all nonadultered (native) samples included in this study cohort and by employing the aforementioned analytical setup. Table 1 and Figure 2 contain proposed candidate peptides based on the following analytical findings: (non)­detectability in treated or untreated condition; group-wise median abundance above 10^5^ cps before or after adulteration to maintain robust and reproducible analytical detectability, and acceptable margins of error for CV across individual samples. KSQPM[15.9949]­GLWR, QVEGM[15.9949]­EDWK, SDVM[15.9949]­YTDWK, and TYM[15.9949]­LAFDVNDEK exhibited single oxidation events of methionine in their primary amino acid sequences only in adulterated samples. Dioxidation of methionine was found, for example, in M[31.9898]­FLSFPTTK, which could also only be observed in adulterated samples. Other than methionine, oxidations observed on tryptophan (LLVVYPW­[15.9949]­TQR) and lysine (QVLDNLT­MEK[15.9949]) were found, too. These types of peptides could be ideal biomarker candidates for detecting oxidatively adulterated samples, as their presence could indicate a non-natural, nonphysiological state of the urine sample in question (Figure 2A). Curiously, we also found examples of unmodified (native) peptides exclusively after oxidative treatment: FSGSILGNK, EENFYVDETTVVK, and GQTLLAVAK, although currently we cannot explain these findings biochemically.

**Figure 2:**
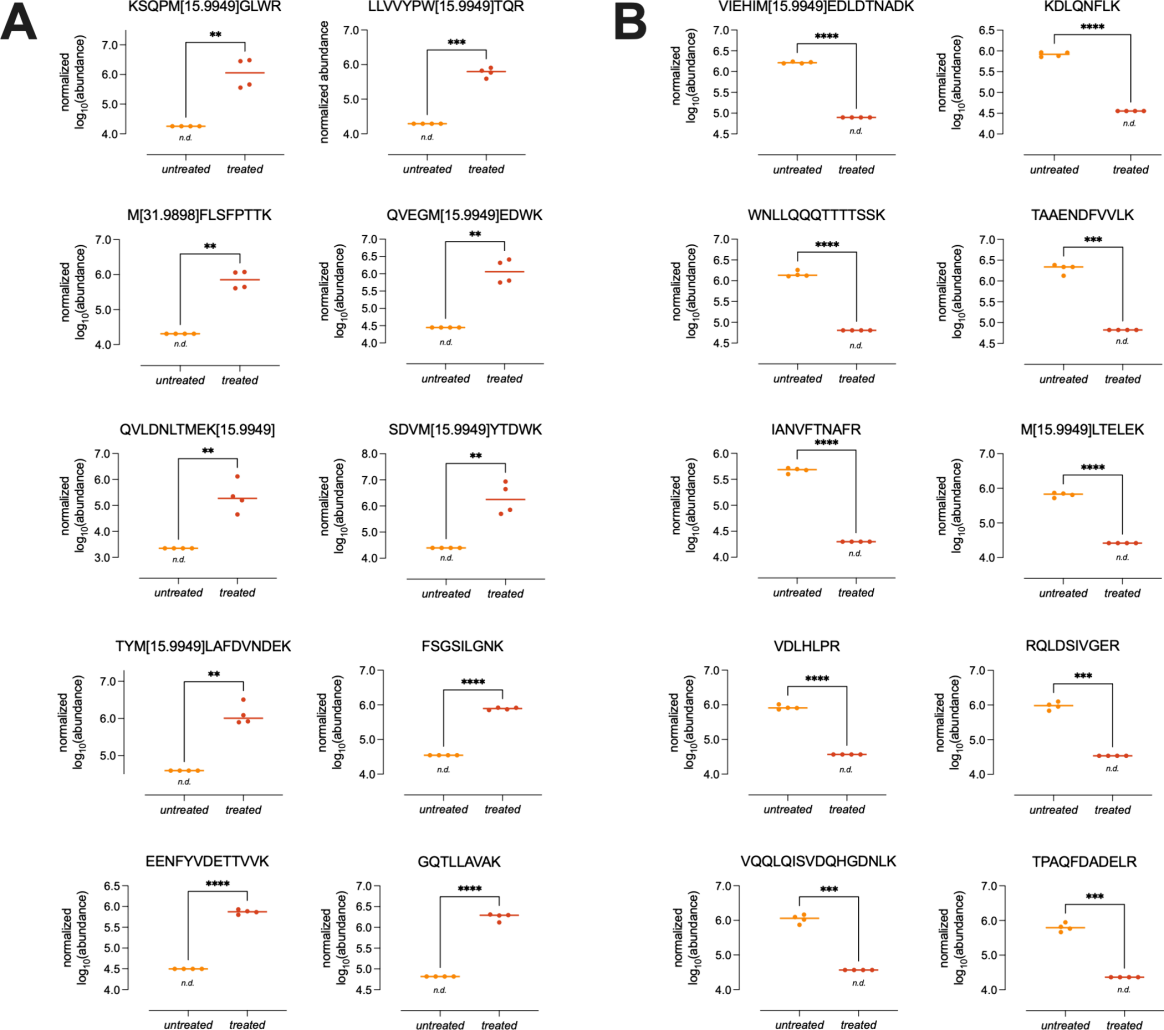
Normalized and log_10_-transformed abundance scatterplots for selected peptides only detectable *after* oxidative treatment (A) and for peptides only detectable before oxidative treatment (B) in urine samples. Horizontal bar represents the median. Paired *t* test applied for significance testing (*, *p* ≤ 0.05; **, *p* ≤ 0.01; ***, *p* ≤ 0.001; ****, *p* ≤ 0.0001).

**Table 1:** Prospective Candidate Peptides for Detecting Oxidative Adulteration of Urine Samples^a^

			untreated		H_2_O_2_-treated		
peptide	protein (ID)	modification	abundance	CV, %	abundance	CV, %	*p* (paired *t* test)
KSQPM[15.9949]GLWR	Zinc-alpha-2-glycoprotein (sp|P25311|ZA2G_HUMAN)	Met oxidation	n.d.	n.a.	1640919	87.4	0.0056
LLVVYPW[15.9949]TQR	Hemoglobin subunit beta (sp|P68871|HBB_HUMAN)	Trp oxidation	n.d.	n.a.	628268	28.5	0.0002
M[31.9898]FLSFPTTK	Hemoglobin subunit alpha (sp|P69905|HBA_HUMAN)	Met dioxidation	n.d.	n.a.	793190	53.6	0.0182
QVEGM[15.9949]EDWK	Zinc-alpha-2-glycoprotein (sp|P25311|ZA2G_HUMAN)	Met oxidation	n.d.	n.a.	1356604	69.9	0.0025
QVLDNLTMEK[15.9949]	Keratin, type I cytoskeletal 9 (sp|P35527|K1C9_HUMAN)	Lys oxidation	n.d.	n.a.	191219	135.4	0.0072
SDVM[15.9949]YTDWK	Alpha-1-acid glycoprotein 2 (sp|P19652|A1AG2_HUMAN)	Met oxidation	n.d.	n.a.	2566371	107.2	0.0082
TYM[15.9949]LAFDVNDEK	Alpha-1-acid glycoprotein 1 (sp|P02763|A1AG1_HUMAN)	Met oxidation	n.d.	n.a.	1028156	75.7	0.0017
FSGSILGNK	Immunoglobulin lambda (sp|A0A075B6I0|LV861_HUMAN)		n.d.	n.a.	778760	7.7	<0.0001
EENFYVDETTVVK	Corticosteroid-binding globulin (sp|P08185|CBG_HUMAN)		n.d.	n.a.	748265	12.3	<0.0001
GQTLLAVAK	Leucine-rich alpha-2-glycoprotein (sp|P02750|A2GL_HUMAN)		n.d.	n.a.	1953940	18.5	<0.0001
VIEHIM[15.9949]EDLDTNADK	Protein S100-A9 (sp|P06702|S10A9_HUMAN)	Met oxidation	1627672	4.7	n.d.	n.a.	<0.0001
KDLQNFLK	Protein S100-A9 (sp|P06702|S10A9_HUMAN)		831613	12.8	n.d.	n.a.	<0.0001
WNLLQQQTTTTSSK	Keratin, type II cytoskeletal 4 (sp|P19013|K2C4_HUMAN)		1354684	16.8	n.d.	n.a.	<0.0001
TAAENDFVVLK	Keratin, type II cytoskeletal 4 (sp|P19013|K2C4_HUMAN)		2178417	23.5	n.d.	n.a.	0.0001
IANVFTNAFR	Myeloperoxidase (sp|P05164|PERM_HUMAN)		485086	11.3	n.d.	n.a.	<0.0001
M[15.9949]LTELEK	Protein S100-A8 (sp|P05109|S10A8_HUMAN)	Met oxidation	675424	14.6	n.d.	n.a.	<0.0001
VDLHLPR	Serpin B3 (sp|P29508|SPB3_HUMAN)		6547342	46.3	n.d.	n.a.	<0.0001
RQLDSIVGER	Keratin, type II cytoskeletal 6A (sp|P02538|K2C6A_HUMAN)		961017	24.9	n.d.	n.a.	0.0001
VQQLQISVDQHGDNLK	Keratin, type II cytoskeletal 4 (sp|P19013|K2C4_HUMAN)		1152911	27.1	n.d.	n.a.	0.0002
TPAQFDADELR	Annexin A1 (sp|P04083|ANXA1_HUMAN)		618393	27.4	n.d.	n.a.	0.0001

a Normalized abundance calculated as median. n.d.: not detected. n.a.: not applicable. CV: coefficient of variation in percent. Number in brackets within peptide sequence on first column refers to *m*/*z* shift due to modification(s) of the amino acid.

On the other hand, we also found mono-oxidized peptides, e.g., VIEHIM[15.9949]­EDLDTNADK and M[15.9949]­LTELEK, only in nonadulterated urine samples (Figure 2B). One possible explanation could be that these peptides are already rather sensitive to baseline (or artifactual) methionine oxidation and tend to further oxidize or even fully degrade after oxidative sample treatment with H_2_O_2_ and were therefore only found in our untreated sample cohort.^36^

Lastly, a rather large number of unmodified peptides could only be measured in nonadulterated samples, for example: KDLQNFLK, WNLLQQQTTTTSSK, TAAENDFVVLK, IANVFTNAFR (Figure 2B) These peptides could be used as a kind of “negative control”; they should be observable if the sample is valid and has not been tampered with, while their possible nondetectability could give rise to suspecting a sample as adulterated. Caution is to be advised, though, as nondetectability can depend on several other influencing factors, for example, changes in (patho-)­physiology, instrumental sensitivity/chromatography, or sample preparation. All of these peptides have in common that they originate from high-abundance proteins (in vivo) and were therefore observed with high abundances and also with acceptable CVs (Table 1). That should also facilitate further downscaling analysis to introduce more rapid and straightforward routine methods of their detection.

### Oxidative Changes on the Hair Proteome: Selection of Potential Adulteration Markers

As analog to the urine sample section above, oxidatively treated hair samples exhibit similar, stark group clustering according to their treatment regimen in multivariate statistical analysis. In the PCA (Figure 3A), cluster separation is not fully achieved, yet a relationship between the extent of oxidative treatment and sample clustering is clearly observed. A pairwise PERMANOVA was performed, and the respective results are shown in Figure (Figure 3B). Based on these, hair treated with 12% H_2_O_2_ can be discriminated from untreated hair samples (R2 0.966), from treated samples with 3% H_2_O_2_ (R2 0.953) and 6% (R2 0.951), while discrimination power diminishes with samples treated with 9% H_2_O_2_ (R2 0.685). A PLS–PA plot roughly paints the same picture: class separation follows a trend depending on the level of oxidative treatment, while complete class separation among all classes was not possible. With the inclusion of two components in PLS-DA, an accuracy of 0.62 was achieved with an R2 of 0.962 and a Q2 of 0.856. Hierarchical clustering was also performed (Figure 3C), illustrated with the top 50 peptides based on their ANOVA score for class clustering. Peptides driving class separation were clustered, highlighted, and annotated to their respective sample group for further downstream investigation.

**Figure 3:**
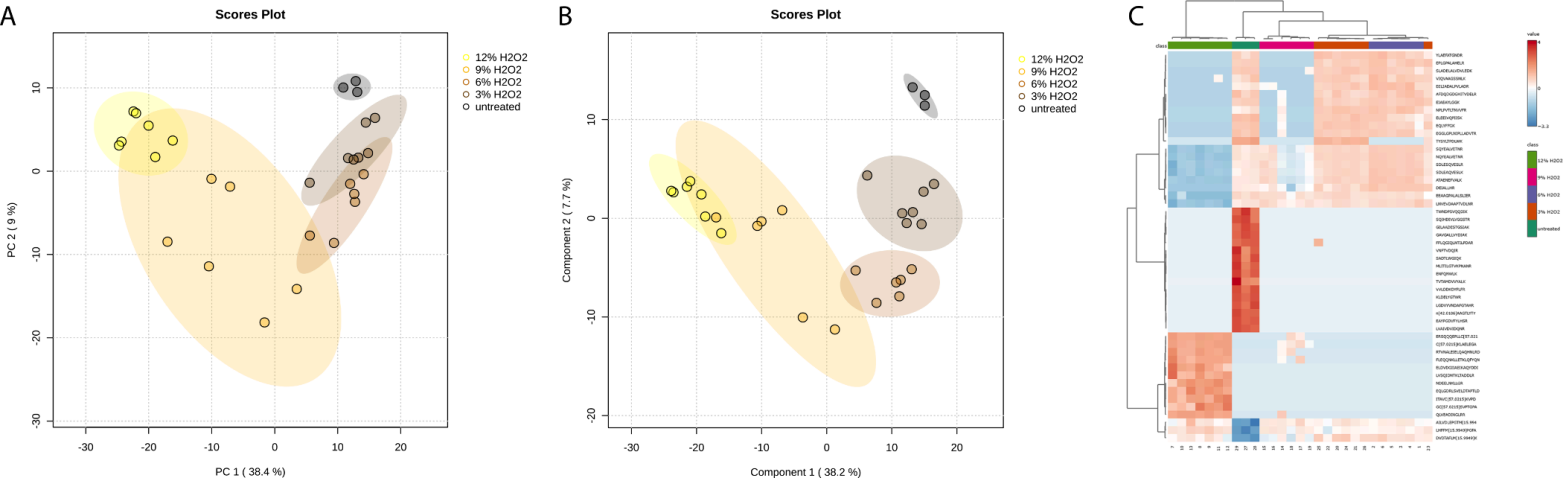
(A) Principal component analysis (PCA) plot of untreated hair samples and samples treated with increasing concentrations of hydrogen peroxide. Pairwise PERMANOVA validation results for group clustering/sample class allocation. (B) PLS-DA plot of untreated hair samples (black) and hair samples treated with increasing concentrations of hydrogen peroxide (color palette). Cross-validation results with two components: Accuracy 0.62; R2 0.962; Q2 0.856. With three components: Accuracy 0.63; R2 0.991; Q2 0.900. (C) Hierarchical clustering representing the top 50 peptides for classification according to ANOVA scores across all 5 sample groups and with sample class clustering enabled. Color gradient (blue to red) represents log_10_-transformed peptide abundances (low to high).

Table 2 and Figure 4 summarize selected peptides that can be used as potential biomarkers for the detection of oxidatively treated (or damaged) hair. These peptides were selected due to their observed pattern before or after treatment: peptides exhibiting detectability either only before or after oxidative treatment were considered ideal candidates, as they would allow for a binary (adulterated/not adulterated) interpretation. Some of the peptides listed in Figure 4 could only be observed after high levels of H_2_O_2_ treatment, while others showed proportional abundances depending on the level of oxidative treatment. Unsurprisingly, the majority of possible biomarker peptides for oxidative hair adulteration are peptides carrying one or multiple oxidations in their amino acid sequence. Tri-(DLNM[47.9847]­DCI­IAEIKAQY­DDVASR, DLNM[47.9847]­DCIIAEIKA­QYDDIVTR) and few dioxidations of methionine (SDLEAQM[31.9898]­ESLKEELL­SLKQNHE­QEVNTLR) as well as oxidations of lysine (LTAEVENAK[15.9949]­CQNSK[15.9949]­LEAAVAQ­SEQQGEA­ALSDAR and SDLEAQVE­SLK[31.9898]­EELLCLKQ­NHEQEV­NTLR) were only observed under harsh oxidative treatment (9–12% H_2_O_2_). Other methionine dioxidation (ETM[31.9898]­QFLNDR) and mono-oxidation (AKQDM[15.9949]­ACLIR) could be detected under all conditions but the untreated hair samples. Other examples of monooxidized (LHFFM[15.9949]­PGFAPLTSR and SKCEEM­[15.9949]­K) and unmodified peptides (NQYEALVETNR) exhibited clear abundance trends in dependence on increasing H_2_O_2_-concentrations (Table 2 and Figure 4B).

**Figure 4:**
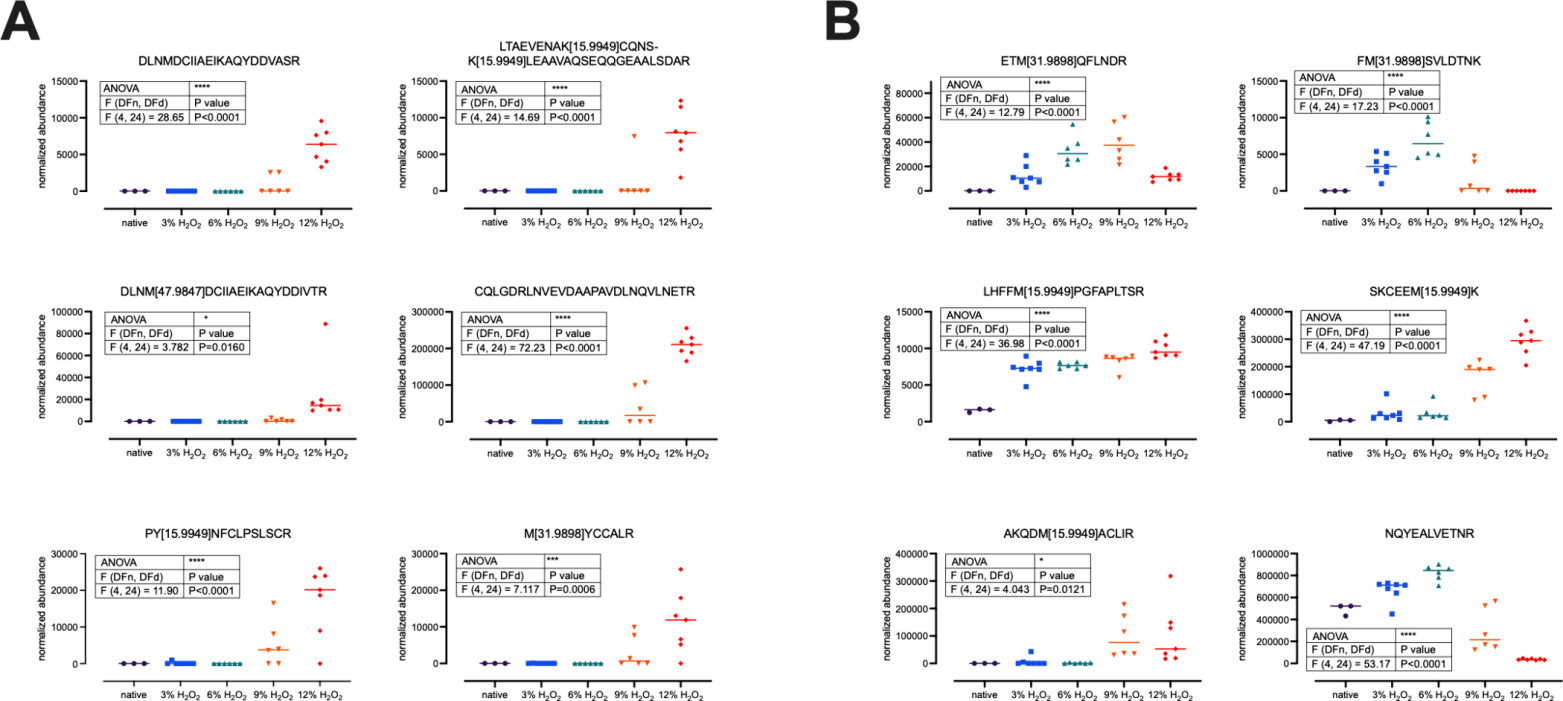
(A) Abundance scatterplots for selected peptides only detectable after strong oxidative treatment of human hair samples. (B) Abundance scatterplots for various selected peptides across different treatment regimens of human hair samples. Normalized and log_10_-transformed abundances are represented in each plot. Horizontal bar represents median. One-way ANOVA results are represented in the respective table of each subplot (*, *p* ≤ 0.05; **, *p* ≤ 0.01; ***, *p* ≤ 0.001; ****, *p* ≤ 0.0001).

**Table 2:** Candidate Peptides for the Detection of Hair Oxidation^a^

			median abundance [CV %]				
peptide	protein origin	modification	untreated	3%	6%	9%	12%
DLNM[47.9847]DCIIAEIKAQYDDVASR	Keratin, type II cuticular Hb5 (sp|P78386|KRT85_HUMAN)	Met trioxidation	n.d.	n.d.	n.d.	n.d.	7382 [98.62%]
DLNM[47.9847]DCIIAEIKAQYDDIVTR	Keratin, type II cuticular Hb6(sp|O43790|KRT86_HUMAN)	Met trioxidation	n.d.	n.d.	n.d.	n.d.	24379 [117.43%]
SDLEAQM[31.9898]ESLKEELLSLKQNHEQEVNTLR	Keratin, type I cuticular Ha3-II (sp|Q14525|KT33B_HUMAN)	Met dioxidation	n.d.	n.d.	n.d.	11345 [149.81%]	86393 [19.11%]
LTAEVENAK[15.9949]CQNSK[15.9949]­LEAAVAQSEQQGEAALSDAR	Keratin, type II cuticular Hb6 (sp|O43790|KRT86_HUMAN)	Lys oxidation (×2)	n.d.	n.d.	n.d.	1240 [244.95%]	7738[45.81%]
CQLGDRLNVEVDAAPAVDLNQVLNETR	Keratin, type I cuticular Ha3-II(sp|Q14525|KT33B_HUMAN)		n.d.	n.d.	n.d.	39863 [126.39%]	208777 [14.05%]
SDLEAQVESLK[31.9898]EELLCLKQNH­EQEVNTLR	Keratin, type I cuticular Ha3-I (sp|O76009|KT33A_HUMAN)	Lys dioxidation	n.d.			8078 [117.54%]	56108 [104.40%]
ETM[31.9898]QFLNDR	Keratin, type I cuticular Ha3-II (sp|Q14525|KT33B_HUMAN	Met dioxidation	n.d.	12635.39 [70.41%]	33699.59 [30.35%]	39753 [40.24%]	11838 [32.24%]
LHFFM[15.9949]PGFAPLTSR	Tubulin beta-2A chain (sp|Q13885|TBB2A_HUMAN)	Met oxidation	1528.46 [16.43%]	7241.67 [17.43%]	7687.57 [4.93%]	8239 [13.47%]	9933 [11.63%]
AKQDM[15.9949]ACLIR	Keratin, type II cuticular Hb6 (sp|O43790|KRT86_HUMAN)	Met oxidation	n.d.	6870.86 [235.34%]	1394.53 [1.57%]	100851 [78.65%]	103024 [105.18%]
FM[31.9898]SVLDTNK	Protein S100-A3 (sp|P33764|S10A3_HUMAN)	Met dioxidation	n.d.	3443.44 [44.73%]	7000.61 [157.28%]	1555 [140.30%]	n.d.
SKCEEM[15.9949]K	Keratin, type II cuticular Hb5 (sp|P78386|KRT85_HUMAN)	Met oxidation	3854.71 [87.66%]	31282.33 [102.59%]	33267.03 [18.57%]	161523 [38.13%]	293693 [17.79%]
NQYEALVETNR	Keratin, type I cuticular Ha3-II (sp|Q14525|KT33B_HUMAN)		491553.42 [10.54%]	664669.11 [14.96%]	826932.94 [23.70%]	299514 [65.62%]	36856 [18.18%]

a Normalized abundance calculated as median. n.d.: not detected. n.a.: not applicable. Number in brackets within peptide sequence on first column refers to *m*/*z* shift due to modification(s) of the amino acid.

Although realistically, full distinction between various, gradual hydrogen peroxide concentrations is not required for real-world application in forensic case-work: distinguishing between “hair sample treated” or “hair sample untreated” (or damaged vs undamaged, respectively) could be sufficient as a sample validity marker, i.e., whether or not the forthcoming forensic hair analysis results for alcohol, pharmacotherapeutics, or drugs of abuse can be correctly interpreted.

## Limitations, Context, and Outlook

### Limitations

As this study aimed to investigate the initial feasibility of the proteomics approach, several open questions remain to be answered and further investigated. Among those, experimental conditions should be broadened in the future in order to better understand the dynamics between oxidative treatment and the resulting findings at the peptide level. H_2_O_2_ only served as an archetype for (oxidative) treatment, but other adulterants are already described in the literature for urine.^11,37,38^ The effect of sample type and condition has yet to be studied in more detail, for example, what role the color and type of hair might play or whether urine pH affects oxidation.

Urine samples in particular should be considered highly heterogeneous (being dependent on hydration, nutrition, physiology, etc.), and although some degree of its heterogeneity might be compensated for by protein normalization during sample preparation, the full extent of its variability remains unexplored within the context of our study. Nonphysiological or pathological conditions need to be taken into account as well, as kidney disease could, for example, have a severe effect on the resulting urinary proteome and thus the analytical findings.

Regarding hair samples, various other sources of oxidative stress or hair damage need to be considered before classifying a suspected hair sample as adulterated: passive UV-light exposure might contribute to overall hair damage and, possibly, oxidation on the protein and peptide level, as well as the use of cosmetic products and treatments such as tinting, (light) bleaching, coloration, permanent curling, hair-straightening, or, perhaps, even the frequent use of a regular hair-drier. Accidental or passive hair damage ideally should be distinguishable from intentional hair adulteration, to circumvent adverse findings. Alternatively, a general, adulteration-agnostic “hair damage index” could be developed based on our findings in order to establish an intrinsic quality control marker for validity testing of forensic hair samples.

### Context: Comparison to Previous Approaches

Compared to previously described approaches within the literature, LC-MS-based proteomics workflows are still relatively new in the forensic communities and usually require different equipment, consumables, instrumental setups, and, of course, expertise. Larger-scale, untargeted approaches may result in deep insights into the studied material but are tedious to plan and execute and are cost- and labor-intensive, too. Previous studies using untargeted metabolomics have demonstrated the feasibility of using small molecules as possible biomarkers to detect adulteration in both hair and urine.^10,11^ While promising, metabolomics strategies are still bottlenecked by procuring confident, confirmatory identification for their reported molecular features, a prerequisite of particular importance, especially in the forensic context. Further, these methods could, in theory, be easily fooled by the manual addition of such biomarker candidates, especially in urine samples. One of the biggest current advantages of proteomics-based approaches is biomarker identification: due to the nature of proteins as biomacromolecules and peptide fragmentation patterns in collision-induced dissociation, identifications can be assigned with extremely high confidence and (usually) without the need for reference standards, resulting in high credibility of the analytical findings.

### Outlook

Distilling such findings from untargeted to targeted MRM-based approaches streamlines the analytical workflow and results in easier integration into pre-existing analytical workflows and infrastructure commonly available in forensic laboratories. By selecting and isolating promising prospect peptide candidates, a targeted MS method could be generated and coupled to a short-gradient, high-flow LC method applicable to routine instrumentation and casework analysis. Another well-reported method of adulterating urine samples is the usage of synthetic, non-human urine or urine from other species in order to circumvent positive drug testing results. In the case of purely synthetic urine, comprehensive untargeted proteomics analyses will reveal the nature of the sample in question by a fundamental lack of protein or, in cases in which protein was added to the synthetic urine, a lack of proteome complexity. For a targeted method, several “validity control” peptide markers could be included to verify the presence of the human urinary proteome. An addition of, for example, bovine serum albumin (BSA) to synthetic urine products could fool authenticity checks based on total protein quantification but would be revealed immediately by conducting a proteome analysis.
